# Impact of Shocks on Mortality in Patients with Ischemic or Dilated Cardiomyopathy and Defibrillators Implanted for Primary Prevention

**DOI:** 10.1371/journal.pone.0063911

**Published:** 2013-05-10

**Authors:** Florian Streitner, Thomas Herrmann, Juergen Kuschyk, Siegfried Lang, Christina Doesch, Theano Papavassiliu, Ines Streitner, Christian Veltmann, Dariusch Haghi, Martin Borggrefe

**Affiliations:** 1st Department of Medicine-Cardiology, University Medical Centre Mannheim, Mannheim, Germany; University of Illinois at Chicago, United States of America

## Abstract

**Background:**

Emerging interest is seen in the paradox of defibrillator shocks for ventricular tachyarrhythmia and increased mortality risk. Particularly in patients with dilated cardiomyopathy (DCM), the prognostic importance of shocks is unclear. The purpose of this study was to compare the outcome after shocks in patients with ischemic cardiomyopathy (ICM) or DCM and defibrillators (ICD) implanted for primary prevention.

**Methods and Results:**

Data of 561 patients were analyzed (mean age 68.6±10.6 years, mean left ventricular ejection fraction 28.6±7.3%). During a median follow-up of 49.3 months, occurrence of device therapies and all-cause mortality were recorded. 74 out of 561 patients (13.2%) experienced ≥1 appropriate and 51 out of 561 patients (9.1%) ≥1 inappropriate shock. All-cause mortality was 24.2% (136 out of 561 subjects). Appropriate shock was associated with a trend to higher mortality in the overall patient population (HR 1.48, 95% CI 0.96–2.28, log rank p = 0.072). The effect was significant in ICM patients (HR 1.61, 95% CI 1.00–2.59, log rank p = 0.049) but not in DCM patients (HR 1.03, 95% CI 0.36–2.96, log rank p = 0.96). Appropriate shocks occurring before the median follow-up revealed a much stronger impact on mortality (HR for the overall patient population 2.12, 95% CI 1.24–3.63, p = 0.005). The effect was driven by ICM patients (HR 2.48, 95% CI 1.41–4.37, p = 0.001), as appropriate shocks again did not influence survival of DCM patients (HR 0.63, 95% CI 0.083–4.75, p = 0.65). Appropriate shocks occurring after the median follow-up and inappropriate shocks occurring at any time revealed no impact on survival in any of the groups (p = ns).

**Conclusion:**

Appropriate shocks are associated with reduced survival in patients with ICM but not in patients with DCM and ICDs implanted for primary prevention. Furthermore, the negative effect of appropriate shocks on survival in ICM patients is only evident within the first 4 years after device implantation.

## Introduction

Treatment with an implantable cardioverter-defibrillator (ICD) improves survival in patients with increased risk for sudden cardiac death due to ventricular tachyarrhythmia (VTA).[1], [2], [3] Although this lifesaving therapy has many benefits, there are emerging data that ICD shocks increase the morbidity and mortality of ICD-patients and are therefore linked to poor clinical prognosis.[4]

In the Multicenter Automatic Defibrillator Implantation Trial II (MADIT II) representing patients with ischemic cardiomyopathy (ICM) due to any history of myocardial infarction and left ventricular dysfunction with a left ventricular ejection fraction (LVEF) ≤30%, single appropriate shock increased the risk of death greater than 3-fold.[5] In the Sudden Cardiac Death in Heart Failure Trial (SCD-HeFT) representing patients with chronic heart failure (NYHA II–III) either due to non-ischemic or ischemic cardiomyopathy and ventricular dysfunction with a LVEF ≤35%, appropriate ICD shocks led to a 3-fold increase in the risk of death.[6]

However, particularly in patients with dilative cardiomyopathy (DCM) and ICDs implanted for primary prevention, the prognostic importance of defibrillator shocks outside the setting of clinical trials is unclear.

## Methods

### Ethics statement and data acquisition

The study complies with the principles expressed in the Declaration of Helsinki. Data were obtained from an existing ICD database at the University Hospital of Mannheim. The database was primarily designed to determine associations between specific blood parameters, occurrence of VTA and clinical prognosis in patients with ICDs and was approved by the local ethical review board. Data stored in the database were collected from clinical records of patients admitted to the hospital or the outpatient clinic. A specific registration number was assigned to each patient entering the database. Patients receiving ICDs for primary prevention were classified into one of the following disease categories: coronary artery disease, dilated cardiomyopathy, hypertrophic cardiomyopathy, arrhythmogenic right ventricular cardiomyopathy, Brugada syndrome, Long-QT syndrome, Short-QT syndrome, others (as e.g. non-compaction cardiomyopathy, amyloidosis or sarcoidosis). Patients with undetermined or uncertain reason for heart failure were assigned to the category ‘others’. For the present analysis, only patients classified as ICM or DCM were selected. The ethics committee waived the need of reapproval or of renewed obtainment of informed consent because of the fact that the presently selected data were analyzed anonymously.

### Patient population

The present study is a prospective longitudinal single center study analyzing data of appropriate and inappropriate ICD therapies and survival in 561 consecutive DCM and ICM patients with ICDs implanted for primary prevention between 1996 and 2008 and known vital status (key date April 2010). To achieve meaningful follow-up data, patients with device implantation after August 2008 were not included into analysis. Eligibility for ICD implantation was based on the international guidelines which may have changed over time.[7], [8]

### Definitions and study endpoints

All patients underwent left heart catheterization before ICD implantation. Coronary artery disease was defined as a stenosis ≥70% in at least 1 major coronary artery or a documented history of myocardial infarction. Diagnosis of DCM was established when dilated cardiac chambers were combined with a LVEF ≤35%. All deaths were classified as cardiac or noncardiac. Deaths classified as noncardiac included vascular events such as a stroke, peripheral arterial embolism, pulmonary embolism and acute hemorrhage and nonvascular events such as those underlying serious lung, liver, kidney or other organ failure, cancer, and sepsis. The death was considered noncardiac even if a VTA occurred but was considered secondary to the underlying noncardiac cause of death.[9] All-cause mortality, shock therapy and end of follow-up were considered as study endpoints.

### Follow-up and classifications

Patients were followed in the outpatient clinic. Characteristics at baseline and data of trimestrial routine or post shock follow-up visits were recorded. Visits included the assessment of medical history and concomitant medication, physical examination, 12-lead electrocardiogram and telemetry device interrogation. ICD therapies were classified appropriate when they occurred in response to ventricular tachycardia (VT) or ventricular fibrillation (VF). Only patients who survived longer than 24 hours after an ICD shock entered analysis.

### Device interrogation and programming

Implanted systems were manufactured by Biotronik (Berlin, Germany), Medtronic (Minneapolis, MN, USA), Boston Scientific [Natick, MA, USA, formerly CPI, Guidant (St. Paul, MN, USA)], and St. Jude Medical/Ventritex (St. Paul, MN, USA). All devices provided extensive data log information and stored endocardial electrograms. All devices were programmed to store far-field electrograms before the onset of detected episodes to aid in rhythm classification. Electrograms were analyzed by 2 independent observers. Classification of device therapy was based on sudden onset, rate, rate stability, and electrogram morphology of the arrhythmia. Electrical storm (ES) was defined as ≥3 separate VTA events ≤24 h. In patients with ES and more VTA episodes than memory capacity of the specific device, VTA episodes were counted as appropriate episodes due to data log information only if all previous VTA episodes with electrograms were appropriate as well, no ICD malfunction was detected during device interrogation and no history of inappropriate ICD therapy was known in this particular patient. If one of the stored episodes with electrogram revealed inappropriate ICD therapy, VTA episodes without electrogram were not counted.

Devices were uniformly programmed using two detection zones. Three antitachycardia pacing (ATP) attempts (bursts of 8 pulses at 84% of the VT cycle length) followed by shock were programmed in a single VT zone. Standardized numbers of intervals for detection were 18/24 for the initial VF detection and 9/12 for redetection (for VT 16 and 12, respectively). In all devices, only full energy shocks were delivered. The lowest VT detection boundary was programmed based on the rate of inducible VT at the electrophysiologic study or by programming empirical detection rates between 130–180 bpm. The average VT detection rate was 167 bpm. VF detection was uniformly programmed at 214 bpm. In the case of VTA faster than 214 bpm, device shocks were the initial therapy. Supraventricular tachycardia discriminators were enabled.

### Statistical analysis

Categorical data are presented as absolute numbers and percentages of the group, continuous variables as mean ± SD. Comparison of normally distributed continuous variables was performed by 2-tailed Student *t-* test. Mann-Whitney test was used for variables with skewed distribution. Chi-square statistics including the chi-square test for trend were used for discrete variables. Follow-up started after implantation of the device and ended at ICD shock therapy, death, or latest follow-up examination. The effect of appropriate and inappropriate device therapy on mortality was analyzed within three patient groups comprising of: 1) all patients, 2) patients with their follow-up ending before or at median follow-up, 3) patients with their follow-up ending after the median follow-up. Differences for ICM and DCM patients were determined.

Survival was graphically displayed according to the method of Kaplan and Meier with comparisons of cumulative survival rate by the log-rank test. Device therapy effects were characterized by calculating hazard ratios (HR) and associated 95% confidence intervals (CI) derived from Cox models. To evaluate potential confounding factors on the diagnosis of mortality, multivariable Cox regression analyses with backward elimination were performed. The models consisted of mortality as the dependent binary variable and defibrillator shocks and other potential confounding factors as independent variables. Two-sided *P* values <0.05 were considered statistically significant. Power analysis was performed using GraphPad StatMate version 2.00 (GraphPad Software Inc., San Diego, CA, USA). All other statistical analyses were conducted using SPSS 19.0 statistical software (SPSS Inc., Chicago, IL, USA) or InStat 3.00 (GraphPad Software Inc., San Diego, CA, USA).

## Results

### Baseline characteristics of the study population

A total of 561 patients with ICD implanted for primary prevention were followed for an average of 55.4±32.7 months (median 49.3). 415 patients suffered from ICM (74%) and 146 from DCM (26%). Most subjects were males (82.9%). Overall mean LVEF was 28.6±7.3%. Baseline characteristics of the patient population are outlined in table 1.

**Table 1:** Baseline characteristics of the study population.

	n = 561
Age (years)	68.6±10.6
Male gender	465 (82.9%)
Coronary artery disease	415 (74%)
Dilative cardiomyopathy	146 (26%)
LVEF (%)	28.6±7.3
NYHA-class III-IV	217 (38.7%)
Atrial fibrillation	206 (36.7%)
History of syncope	123 (21.9%)
Diabetes mellitus	90 (16%)
COPD	107 (19.1%)
Serum creatinine >1.3 (mg/dl)	174 (31%)
LBBB	194 (34.6%)
Single-chamber ICD	362 (64.2%)
Dual-chamber ICD	136 (24.2%)
CRT-D	63 (11.2%)
ß-blockers	476 (84.8%)
ACE-inhibitors or ARBs	514 (91.6%)
Amiodarone	52 (9.3%)
Digitalis glycosides	183 (32.6%)
Diuretics	396 (70.6%)
Statins	379 (67.9%)

Data are presented as the mean value ± SD for continuous variables and number (percentage) for categorical variables. ACE  =  angiotensin converting enzyme; ARB  =  angiotensin receptor blockers; COPD = chronic obstructive pulmonary disease, CRT-D  =  cardiac resynchronisation therapy – defibrillator; ICD  =  implantable cardioverter-defibrillator; LBBB  =  left bundle branch block; LVEF  =  left ventricular ejection fraction; NYHA  =  New York Heart Association.

Patients with DCM were significantly more often female (p = 0.041), were significantly younger (p<0.001), had a lower mean LVEF (p<0.001) and were more often classified in NYHA class III-IV (p = 0.004). These and other significant differences in baseline characteristics comparing patients with ICM and DCM are outlined in table 2.

**Table 2:** Significant differences in baseline characteristics between ICD patients with ICM and DCM.

	ICM (n = 415)	DCM (n = 146)	p-value
Age (years)	69.8±9.7	65.1±12.2	<0.001
Male gender	352 (84.8%)	113 (77.4%)	0.041
NYHA class III-IV	146 (35.2%)	71 (48.6%)	0.004
LVEF (%)	29.3±7.1	26.4±7.4	<0.001
Atrial fibrillation	169 (40.7%)	37 (25.3%)	0.001
Single chamber ICD	275 (66.3%)	87 (59.6%)	0.002
Digitalis glycosides	120 (28.9%)	63 (43.2%)	0.002
Diuretics	279 (67.2%)	117 (80.1%)	0.003
Statins	332 (80%)	47 (32.3%)	<0.001

Data are presented as the mean value ± SD for continuous variables and number (percentage) for categorical variables. DCM  =  dilated cardiomyopathy; ICD  =  implantable cardioverter-defibrillator; ICM  =  ischemic cardiomyopathy; LVEF  =  left ventricular ejection fraction; NYHA  =  New York Heart Association.

### All-cause and cardiac mortality

Overall mortality during follow-up was 24.2% (136 out of 561 subjects). Within comparable follow-up durations (p = 0.989), no statistical difference was determined with regard to all-cause (106 out of 415 (25.5%) for ICM vs. 30 out of 146 for DCM (20.5%), p = 0.226) or cardiac mortality (60 out of 106 (56.6%) for ICM vs. 22 out of 30 (73.3%) for DCM, p = 0.098). No significant differences were determined when calculating mean time to death after device implantation (38.2±33 months for ICM vs. 42.9±37.2 for DCM, p = 0.505). Variables associated with death in univariate analysis were history of (p = 0.013) and number of VF events (p = 0.008), history of electrical storm events (ES) (p = 0.008), LVEF <30% (p = 0.001), older age (p = 0.04), New York Heart Association (NYHA) class III-IV (p = 0.007), history of syncope (p = 0.006), history of atrial fibrillation (p = 0.002), presence of diabetes mellitus (p<0.001) or of left bundle branch block (p = 0.007), digitalis intake (p = 0.01) and treatment with statins (p = 0.007).

### Appropriate device therapy

**Figure 1:**
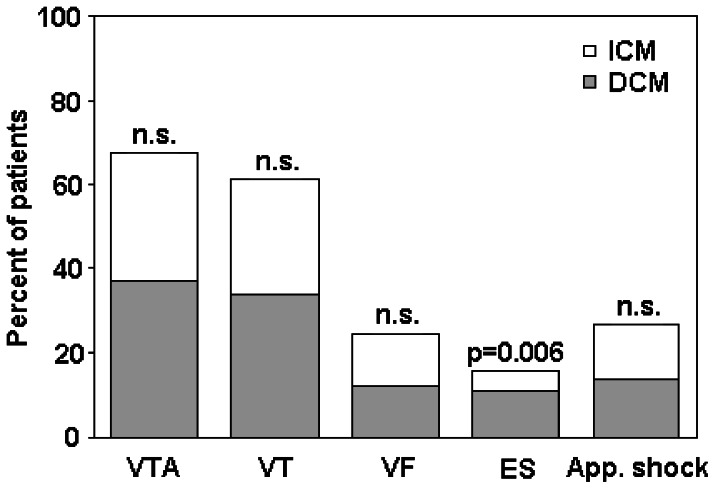
Comparison of VTA episodes and appropriate shocks in patients with DCM and ICM. app. shock  =  appropriate shock; DCM  =  dilated cardiomyopathy; ES  =  electrical storm; ICM  =  ischemic cardiomyopathy; VF  =  ventricular fibrillation; VT  =  ventricular tachycardia; VTA  =  ventricular tachyarrhythmia.

A total of 2153 appropriate VTA episodes occurred during follow-up with 1970 potentially ATP-terminable episodes being treated in the VT and 184 episodes in the VF detection zone. At least one VTA with appropriate device therapy was detected in 181 out of 561 patients (127 out of 415 patients with ICM (30.6%) vs. 54 out of 146 patients with DCM (37%), p = 0.156). At least one appropriate shock was delivered in 74 out of 561 patients (54 out of 415 patients with ICM (13%) vs. 20 out of 146 patients with DCM (13.7%), p = 0.833). Patients with ICM received a total of 130 appropriate shocks (mean 0.3±1.1), whereas patients with DCM received a total of 67 appropriate shocks (mean 0.4±2, p = 0.294). In the overall patient population, a significant difference in age was determined between patients with or without appropriate shock before median follow-up (p = 0.035, for ICM patients p = 0.051 respectively). During follow-up, 27.5% of the ICM patients and 33.6% of the DCM patient population experienced ≥1 VT episode (p = 0.163) and 12.3% of both patient populations experienced VF episodes (p = 0.99). ES occurred more frequently in DCM patients (16 out of 146 patients (11%) vs. 19 out of 415 patients (4.6%), p = 0.006). Figure 1 delineates the distribution of VTA episodes and delivered appropriate shocks in patients with DCM and ICM.

When comparing ICM and DCM patients, mean number of VT episodes (2.77±12.8 for ICM vs. 5.6±23.5 for DCM, p = 0.167), of VF episodes (0.29±1.2 for ICM vs. 0.43±2.0 for DCM, p = 0.306), of ES episodes (0.15±0.9 for ICM vs. 0.25±1.0 for DCM, p = 0.28), of time to first appropriate therapy after device implantation (17.3±27.7 months for ICM vs. 17.6±32.6 for DCM, p = 0.937) as well as mean VT cycle length (331±32 ms for ICM vs. 326±29 ms for DCM, p = 0.396) did not differ significantly.

### Impact of appropriate device therapy on mortality

Appropriate ATP therapy alone had no significant impact on all-cause mortality when comparing event rates of dead vs. alive patients in the overall patient population or within subgroups of patients with ICM or DCM (p = ns). Univariate analysis revealed that patients who died during follow-up significantly more often experienced ≥1 appropriately shocked VTA episode compared to survivors (27 out of 136 patients (19.9%) vs. 47 out of 425 patients (11.1%), p = 0.011). Moreover, a significant trend towards elevated mortality was found with increasing numbers of appropriate shocks during follow-up (chi-squared test for trend p = 0.025).

The observation that more shocks occurred in patients who died during follow-up was driven by the ICM patient subgroup (20.8% dead patients with shocks vs. 10.7% shocks in survivors, p = 0.008). DCM patients did not reveal a significant difference within subgroups (16.7% dead patients with shocks vs. 12.9% shocks in survivors, p = 0.056).

### Inappropriate device therapy

Inappropriate device therapy of any kind occurred in 80 out of 561 patients (14.3%). Inappropriate shocks (with or without ATP) were delivered in 51 out of 561 patients (9.1%). No significant association between the occurrence of inappropriate and appropriate shocks was determined within the overall patient population or within DCM or ICM patients (p = ns).

Patients with DCM were more likely to receive an inappropriate ICD therapy of any kind (31 out of 146 patients (21.2%) with DCM vs. 49 out of 415 patients (11.8%) with ICM (p = 0.005), HR = 2.01 (95% CI 1.2–3.3), p = 0.009). As the incidence of inappropriate shocks was comparable within the groups (18 out of 146 patients (12.3%) with DCM vs. 33 out of 415 patients (8%) with ICM, p = 0.31), the higher burden of inappropriate device therapies of any kind are explained by a higher proportion of DCM patients receiving inappropriate ATP therapy (22 out of 146 patients (15.1%) with DCM vs. 25 out of 415 patients (6%) with ICM (p = 0.001), HR  = 2.77 (95% CI 1.5–5.1), p = 0.002).

The same pattern was determined when comparing the occurrence of inappropriate device therapies of any kind and the occurrence of inappropriate ATP therapy after median follow-up (for device therapies of any kind: 23 out of 70 patients (32.9%) with DCM vs. 32 out of 210 patients (15.2%) with ICM (p = 0.001), HR = 2.72 (95% CI 1.5 – 5.1), p = 0.003; for inappropriate ATP therapy: 16 out of 70 patients (22.9%) with DCM vs. 16 out of 210 patients (7.6%) with ICM (p = 0.001), HR = 3.59 (95% CI 1.7 – 7.7), p = 0.002). Before median follow-up, the incidence of inappropriate ICD therapy of any kind did not differ significantly within the two groups (p = ns). The incidence of inappropriate shocks, delivered alone or in conjunction with ATP, was comparable within the groups at any time of the study (p = ns).

### Impact of inappropriate device therapy on mortality

Inappropriate device therapy of any kind revealed no significant impact on mortality in the overall patient population or in patients with ICM, neither within the overall follow-up nor before or after median follow-up (p = ns). When comparing the incidence of any inappropriate device therapy in patients with DCM before or after median follow-up, no significant differences were determined (p = ns). During the overall follow-up, inappropriately delivered ATP was associated with higher mortality in DCM patients (9 out of 30 patients dying during follow-up vs. 13 out of 116 patients alive, p = 0.1), HR 3.4 (95% CI 1.3 – 9), p = 0.02), whereas inappropriately delivered shocks (with or without ATP) revealed no impact on mortality in this patient population (p = 0.15). According to the expectations, the power to detect significant differences in mortality after inappropriate device therapy within compared patient populations was low whenever differences in mortality were smaller than 5 percentage points (as e.g. marginal 1.4 percentage points higher all-cause mortality in the overall patient population after inappropriate shock therapy compared to patients without inappropriate shocks).

### Kaplan-Meier survival estimation and risk models

**Figure 2:**
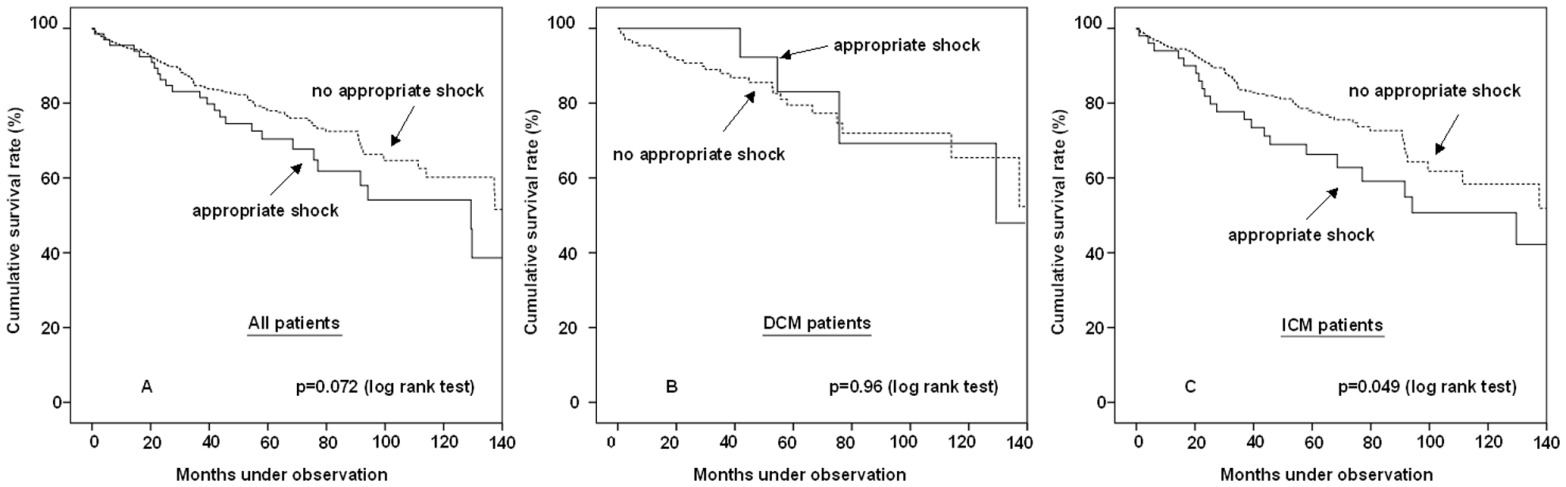
A-C. Kaplan Meier survival estimation after appropriate shocks (complete follow-up). A significant association between appropriate shocks and survival is only determined in patients with ICM.

**Figure 3:**
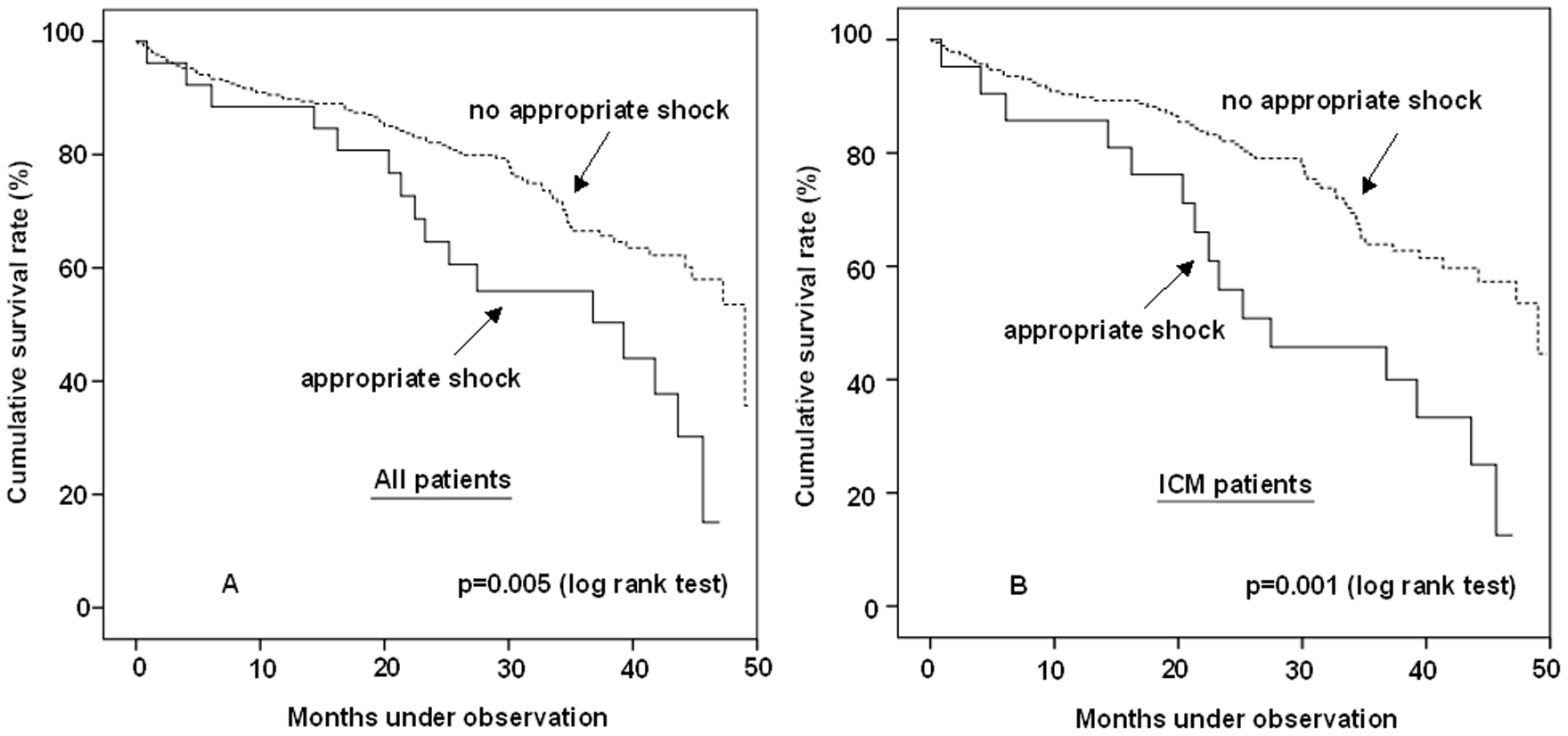
A-B. Kaplan Meier survival estimation after occurrence of appropriate shocks before median follow-up. Kaplan Meier Curves displaying that the significant effect of appropriate shocks on survival in the overall patient population is primarily driven by the ICM subgroup.

Kaplan Meier survival estimation and hazard ratios derived from Cox models determined a trend to an increased risk of death after ≥1 appropriate shock therapy vs. no shock therapy in the overall patient population (HR 1.48, 95% CI 0.96–2.28, log rank p = 0.072, fig. 2A). In contrast to DCM patients (HR 1.03, 95% CI 0.36–2.96, log rank p = 0.96, fig. 2B), appropriate shocks were associated with an increased risk of subsequent death in patients with ICM (HR 1.61, 95% CI 1.00–2.59, log rank p = 0.049, fig. 2C). When performing survival estimation of the 281 out of 561 patients (50%) who reached the study endpoint before the median follow-up of 49.3 months, the impact of appropriate shock therapy on mortality reached high significance levels in the overall patient population (16 out of 30 patients (51.6%) with appropriate shocks died versus 79 out of 251 patients (31.5%) without appropriate shocks, HR 2.12, 95% CI 1.24–3.63, p = 0.005, fig. 3A). The effect was driven by the ICM subgroup (15 out of 24 patients (62.5%) with appropriate shocks died versus 62 out of 181 patients (34.3%) without appropriate shocks, HR 2.48, 95% CI 1.41–4.37, p = 0.001, fig. 3B), as appropriate shock therapy again did not influence survival of DCM patients (1 out of 6 patients (16.7%) with appropriate shocks died versus 18 out of 76 patients (23.7%) without appropriate shocks, HR 0.63, 95% CI 0.083–4.75, p = 0.65).

Appropriate shocks occurring later than the median follow-up of 49.3 months did not reveal a significant association with reduced survival in the overall patient population (11 out of 44 patients (25%) with appropriate shocks died versus 30 out of 236 (12.7%) patients without appropriate shocks, log rank p = 0.26) or in both subgroups (7 out of 30 ICM patients (23.3%) with appropriate shocks died versus 22 out of 180 patients (12.2%) without appropriate shocks, log rank p = 0.52 and 4 out of 14 DCM patients (28.6%) with appropriate shocks died versus 8 out of 56 patients (14.3%) without appropriate shocks, log rank p = 0.30). In order to exclude that the power was insufficient to detect a decrease in survival within the DCM patient population, a power analysis was performed. The calculated power was 85% with a significance level of 0.05 and was therefore sufficient.

In multivariate Cox regression models, appropriate shock remained tending to be associated with all-cause mortality in the overall patient population (HR 1.65, 95% CI 0.95–2.9, p = 0.075) and remained significantly associated with all-cause mortality in the ICM patient subgroup (HR 1.99, 95% CI 1.12–3.54, p = 0.019) even after adjustment for age and other independent variables like gender, beta-blocker use or NYHA-class. Patients with DCM revealed no significant association between appropriately delivered shocks and all-cause mortality (HR 0.94, 95% CI 0.32–2.8, p = 0.92) within this model. When analyzing a Cox regression model with additional independent variables like statin medication, digitalis intake, diuretics intake and presence of atrial fibrillation, appropriate shocks again were not significantly associated with mortality in DCM patients (HR 0.83, 95% CI 0.27–2.6, p = 0.75) but remained tending to be significantly associated with mortality in ICM patients (HR 1.55, 95% CI 0.95–2.5, p = 0.08). Inappropriate ICD shock added to the Cox regression models as an additional independent variable was not associated with all-cause mortality in any of the groups at any time (p = ns).

## Discussion

The present study comprising of 561 ICD recipients for primary prevention confirmed the consistently reproducing relationship between shocked VTA episodes and increased mortality risk. However, we could demonstrate that appropriate shock therapy reduces survival only in patients with ICM but not in patients with DCM. Furthermore, the negative effect of appropriate shocks on survival in ICM patients was only evident within the first 4 years after ICD implantation. Inappropriate ICD shocks were not associated with higher mortality.

The relationship between shocked VTA episodes and mortality, recently proven for patients with ICDs for primary prevention and for secondary prevention [5], [6], [10], was often summarized as follows: patients with VTA and shocks have higher mortality than otherwise similar patients with neither, and patients with more VTA and more shocks have higher mortality than patients with less of both.[4] When considering the results of the present study, this statement would only be valid for ICM patients but not for DCM patients.

To the best of our knowledge, the effect of appropriate shocks on mortality in patients with DCM and ICDs implanted for primary prevention is underrepresented in literature so far. Poole et al. presented data of patients participating in the SCD-HeFT trial enrolling patients with nonischemic and ischemic heart failure.[6] Authors concluded that the occurrence of an appropriate ICD shock was associated with a markedly increased risk of death. In that study, 89 out of 391 patients (22.7%) with nonischemic heart failure experienced ≥1 appropriate shock therapy and 18 patients (4.6%) died during follow-up. A comparable amount of patients with ischemic heart failure received ≥1 appropriate shock therapy during follow-up (93 out of 420 patients, 22.1%), but the proportion of deaths in this patient population was markedly higher (49 out of 420, 11.7%) and the time from shock to death was markedly shorter resulting in a lower one-year survival. With regard to the effect of appropriate shock therapy on mortality, only pooled data including both patient populations were presented. Therefore, it cannot be ruled out that the effect of appropriate shock therapy on mortality was driven by the patient population with ischemic heart failure. This would support the findings of the present study revealing no significant impact of appropriate shocks on mortality when analyzing the data of DCM patients alone.

In the Defibrillator in Acute Myocardial Infarction Trial (DINAMIT), high risk post myocardial infarction (6 to 40 days) patients with an LVEF <35% were randomized to primary preventive ICD recipients or non-recipients.[11] Authors published mortality data of patients with appropriate device therapy occurring within 4 years after randomization (mean follow-up 28±14 months). The 5-fold increased relative risk of death after mostly shock therapy for VTA matched the 3.4-fold increased risk of death after shocks within 3 years after randomization in the MADIT II trial [5] and matches the 2.5-fold increased risk of death after early appropriate shock therapy for ICM patients of the present study.

However, the time-dependant analysis of the present study revealed that appropriate shock therapies occurring later than the median follow-up of 4 years did not develop any significant impact on survival neither in the ICM subgroup nor in the overall or the DCM patient population. An unequivocal explanation for this observation cannot be provided, but it can be assumed that appropriate shocks early after ICD implantation may have a stronger impact on mortality compared to shocks occurring years after device implantation. Dorian et al. noticed that the increase in risk of death soon after receiving ICD shocks suggests that VTA in these patients is associated with a “step” change in the course of the underlying cardiac disease and thus in the prognosis for nonarrhythmic mortality. [11], [12] This aspect is relevant as in DINAMIT and in the Immediate Risk Stratification Improves Survival (IRIS) trial (mean follow-up 37 months) [11], [12], the negative effects of appropriate shocks led to a higher risk of consecutive non-arrhythmic death offsetting the observed sudden cardiac death reduction in the ICD group as a whole.

It is an often held view in clinical trials that shocks are a marker for, but mechanistically unrelated to, the higher mortality and that the lack of a mortality benefit in ICD groups despite VTA electric therapies is not a device failure, but a patient failure.[4] The presence of time dependant interactions between shocked VTA episodes and increased mortality risk in the present study suggests that patient failures occur more often if cardiac ischemia is chronically present or easily inducible, as long-living patients with ICM and patients with DCM and therefore no obvious predisposition for ischemia did not reveal any relationship of appropriate shock therapy and mortality risk at any time of the follow-up. Some other clinical facts support this hypothesis. In DINAMIT, only two third of the high-risk patient population received any form of acute reperfusion therapy.[11] Therefore, patients might have been more susceptible to harm from recurrent myocardial ischemia. In contrast, there is existing evidence that coronary artery bypass grafting, representing a robust revascularization strategy, suppresses inducible VF in cardiac arrest survivors [13], potentially leading to less future cardiac deterioration.

### Clinical implications

Since VF can only be terminated with shocks, promotion of strategies to minimize appropriate shocks is of high priority. Adequate drug treatment, optimal revascularization therapy or otherwise substrate modification can help to suppress VTA and therefore avoid future shock therapy. Additionally, the efficacy of ATP has to be further improved as ATP is the most effective strategy to reduce appropriate shocks.[14], [15] Nonetheless, despite any suggestion that shocks increase risk of death, ICDs prolong survival.[16]

### Limitations

Neither the association between shocks and death in prior studies nor in the present study prove that shocks are causal. Investigations of shock-related myocardial injury have focused on acute effects and may therefore be insufficient to account for the presently observed reduced survival after appropriate shocks. Further studies are needed to assess potential modifications of the hearts signalling pathways by VTA and shocks. It is to mention that the present study was not powered to determine significant differences between compared groups if the differences in mortality rates were below 5 percentage points. Additionally, recommendations for ICD programming have changed over time. Therefore, less numbers of intervals for detection and a lower VF cut-off rate than currently recommended were programmed which might have influenced the incidence of appropriately delivered ICD shocks. To discriminate between DCM and ICM, criteria comparable to those used by the SCD-Heft investigators were applied.[1], [2], [3] Viability studies were not performed in all patients. Therefore, it cannot be excluded that in some patients assigned to the ICM group the severity of cardiomyopathy was out of proportion to the extent of angiographic coronary artery disease.

## Conclusion

The present observation reveals that appropriate shock therapy of defibrillators implanted for primary prevention only reduces survival in patients with ischemic cardiomyopathy if appropriate shocks occur within the first 4 years after device implantation. Shock therapy delivered in patients with stable coronary artery disease over years or in patients with dilative cardiomyopathy is not associated with an increased mortality risk.
